# Secure Transmission of Cooperative Zero-Forcing Jamming for Two-User SWIPT Sensor Networks

**DOI:** 10.3390/s18020331

**Published:** 2018-01-24

**Authors:** Xuanxuan Tang, Yueming Cai, Wendong Yang, Weiwei Yang, Dechuan Chen, Junquan Hu

**Affiliations:** College of Communications Engineering, Army Engineering University of PLA, No. 88 Houbiaoying, Qinhuai District, Nanjing 210007, China; tang_xx@126.com (X.T.); ywd1110@163.com (W.Y.); wwyang1981@163.com (W.Y.); chenchuan927@163.com (D.C.); junquan_hu@163.com (J.H.)

**Keywords:** physical layer security, zero-forcing jamming, secrecy outage probability, secrecy throughput, wireless sensor networks

## Abstract

In this paper, the secrecy performance of the two-user simultaneous wireless information and power transfer (SWIPT) sensor networks is studied and a novel secure transmission scheme of cooperative zero-forcing (ZF) jamming is proposed. The two sensors opportunistically conduct the SWIPT and cooperative ZF jamming, respectively, where the energy required for jamming the eavesdropper is provided by the SWIPT operation so as to keep the energy balance at the sensors in the long run. By deriving the exact closed-form expressions of the secrecy outage probability and the secrecy throughout, we provide an effective approach to precisely assess the impacts of key parameters on the secrecy performance of the system. It has been shown that the secrecy outage probability is a monotonically increasing function of the growth of secrecy rate ($R_{s}$), and a monotonically decreasing function of the increase of the transmit signal-to-noise ratio ($\gamma_{S}$), and energy conversion efficiency (η). Furthermore, the secrecy throughput could be enhanced when η increases, which becomes especially obvious when a large $\gamma_{S}$ is provided. Moreover, the existence of an optimum $R_{s}$ maximizing the secrecy throughput is depicted, which also grows with the increase of $\gamma_{S}$. Simulations are provided for the validation of the analysis.

## 1. Introduction

Because of the broadcast nature of wireless medium, it is a critical issue to secure the transmission in the design of wireless sensor networks [1]. The security is conventionally tackled through higher layer techniques, e.g., cryptographic protocols, which, however, could not guarantee the required security level alone for the large-scale wireless sensor networks due to the significant increase in the complexity of key distribution and management [2]. In contrast to conventional cryptographic approaches conducted at higher layers, the physical layer security (PLS) tries to secure the wireless networks against eavesdropping by exploiting the inherent channel randomness at the physical layer. Hence, PLS has been widely regarded as an effective supplementary protocol for wireless communications and, thus, has gained much attention in research communities [3,4,5].

The main idea of PLS is that, by making and enlarging the capacity differences between the legitimate channels and the wiretapping channels, the information could be transmitted at a predefined secrecy rate so that only the authorized receiver can decode the data while the eavesdropper could not [6]. Various advanced techniques have been proposed to further enhance the potential benefits of PLS, such as antenna selection [7], cooperative relaying [8], cooperative jamming [9], etc. Generally speaking, the security of wireless transmission could be significantly increased if jamming signals could be carefully exploited in the legitimate network. As a consequence, the cooperative jamming has been widely applied in the PLS research in numerous systems, such as single-input-multiple-output (SIMO) networks [10], two-way relay scenarios [11], multiple-input-multiple-output (MIMO) wiretap-channel settings [12], etc.

More recently, the simultaneous wireless information and power transfer (SWIPT) has become an appealing technique in wireless communications due to its great potential in tackling the energy bottleneck issue, especially in some energy-constrained cases [13,14,15]. Authors in [16] investigated the safeguarding approach for SWIPT systems with both the eavesdropper and the friendly jammer harvesting energy from the wireless signals, and the optimal power allocation strategy based on the Lagrange method was then proposed. In [17], a cooperative jamming aided robust secure transmission for SWIPT multiple-input-single-output (MISO) networks was presented, where the objective of the source and the jammer was to maximize the secrecy rate and also supply wireless power to the energy receiver and the destination. However, an additional jammer must be deployed in literature [16,17], which is practically costly in the construction and upgrading of current wireless systems, especially in the wireless sensor networks.

There has been some research investigating the security issue of scenarios where no additional jammer is available and the user harvests the energy and also acts as the jammer itself [18,19,20]. Work [18] studied the secrecy performance of full-duplex SWIPT networks, where the two-antenna user received information and energy with one of its antennae by applying the power-splitting SWIPT protocol, and sent the jamming signal to confuse the eavesdropper with the other antenna. Under a similar system of deployment, the security of the time-switching SWIPT protocol was examined in [19], where the secrecy outage performance and the secrecy energy efficiency were formulated. The author in [20] extended the research in the cognitive networks, where the energy collected by the receiving antenna via the power-splitting SWIPT protocol was used for producing jamming signals by the transmitting antenna. However, the shortage of [18,19,20] is obvious. On the one hand, an effective self-interference cancellation (SIC) method at the users is required to guarantee the validity of the secure schemes, as the users in this literature all work in the full-duplex mode. On the other hand, both the power-splitting and the time-switching approaches applied in these literature will definitely increase the complexity of realization significantly. As a result, the practical value of these schemes in wireless sensor networks is greatly limited.

Motivated by the above observations, we present a novel secure transmission scheme for the proposed two-user SWIPT sensor networks where the two sensors conduct the cooperative zero-forcing (ZF) jamming opportunistically and mutually with the harvested energy. We note that the proposed two-user cooperative pair model is rather practical because each sensor in actual sensor networks can choose another sensor nearby to form this pair and then conduct cooperative jamming in turn. It is also highlighted that the sensors are generally energy-constrained, which again reveals the advantage of the proposed cooperative jamming scheme because it does not consume any energy of the sensors in the long run due to the SWIPT operation. Furthermore, neither the full-duplex and SIC techniques nor the power-splitting and time-switching methods are needed in the proposed scheme, which is of great benefit in practical realization. The remainder of the work is organized as follows: Section 2 characterizes the system model and presents the secure transmission scheme. In Section 3, the exact secrecy analysis of the proposed scheme is carried out. Section 4 conducts the simulations and gives the discussions. Finally, Section 5 summarizes the whole paper.

***Notation*:** Throughout this paper, the boldface uppercase letters are used to denote matrices or vectors. ${\left(\cdot \right)}^{T}$ and ${\left(\cdot \right)}^{H}$ are denoted as the transpose operation and the conjugate transpose operation, respectively. $F_{\gamma }\left(\cdot \right)$ and $f_{\gamma }\left(\cdot \right)$ represent the cumulative distribution function (CDF) and the probability density function (PDF) of random variable γ, respectively. E[·] denotes the expectation operation.

## 2. System Model

### 2.1. System Description

**Figure 1:**
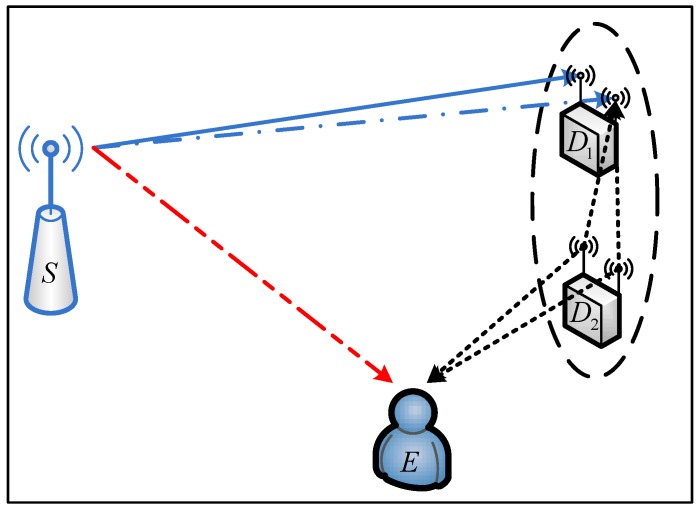
System model.

We consider a downlink SWIPT sensor network as illustrated in Figure 1, which consists of a source node *S*, a pair of two sensor nodes $D_{1}$ and $D_{2}$, and an eavesdropper *E*. All nodes are equipped with a single antenna, except for the two sensors, which both have only two antennae without having to increase too much complexity of realization [19,20]. In addition, we assume that the channel state information (CSI) of the legitimate links is available, while the CSI of the eavesdropping link is not known by the legitimate nodes. This is a typical passive eavesdropping scenario which is more practical than active eavesdropping [21] and has been widely used in existing literature (see [22,23,24] and the references therein). It is also assumed that all the channels between two nodes experience quasi-static Rayleigh fading, such that the channel coefficients keep constant during a packet time $T_{0}$ but vary independently from one packet time to another. Furthermore, all the channels of $S-D_{i,j}$ and $D_{i,j}-E$ are assumed to be independent and identically distributed (i.i.d), respectively.

### 2.2. Secure Transmission

At each slot, the specific antenna of a certain sensor that maximizes the instantaneous transmission channel capacity is chosen for information receiving (IR). At the same time, the remaining antenna of the selected sensor will be assigned for energy harvesting (EH). In addition, the jamming operation is introduced in order to enhance the security of the transmission. More specifically, the cooperative ZF jamming is applied in this paper by the other sensor, so that the IR process is not interfered.

Without loss of generality, we assume that the $D_{i^{*},j^{*}}$ is selected in a certain slot, namely

(1) $$\left(i^{*},j^{*}\right)=\arg\max_{i,j\in \left\{1,2\right\}}\;\left(g_{SD_{i,j}}\right),$$

where $g_{\left(.\right)}=\left|h_{\left(.\right)}^{2}\right|$ , and $h_{SD_{i,j}}$ represents the channel coefficient between *S* and the *j*-th antenna of *i*-th sensor. At the same time, the antenna $D_{i^{*},3-j^{*}}$ is allocated for collecting energy, and the other sensor $D_{3-i^{*}}$ is assigned to produce ZF jamming signals in order to increase the security of the transmission. As described above, we can summarize the working mode of all the antennae when $D_{i^{*},j^{*}}$ is determined, which is shown in Table 1 for the better readability.

As a result, the signals for IR and EH can be given by Labels (2) and (3), respectively,

(2) $$y_{D_{i^{*},j^{*}}}=\sqrt{P_{J}}h_{D_{3-i^{*}}D_{i^{*},j^{*}}}^{H}w_{ZF}x_{J}+\sqrt{P_{S}}h_{SD_{i^{*},j^{*}}}x_{S}+n_{D_{i^{*},j^{*}}},$$

(3) $$y_{D_{i^{*},3-j^{*}}}=\sqrt{P_{J}}h_{D_{3-i^{*}}D_{i^{*},3-j^{*}}}^{H}w_{ZF}x_{J}+\sqrt{P_{S}}h_{SD_{i^{*},3-j^{*}}}x_{S}+n_{D_{i^{*},3-j^{*}}},$$

where $P_{S}$ and $P_{J}$ represent the transmit power of *S* and the jamming power of sensors, $h_{D_{3-i^{*}}D_{i^{*},j^{*}}}$, $h_{D_{3-i^{*}}D_{i^{*},3-j^{*}}}\in C^{2\times 1}$ represent the channel coefficient vectors from $D_{3-i^{*}}$ to $D_{i^{*},j^{*}}$ and $D_{i^{*},3-j^{*}}$, respectively. $x_{S}$ and $x_{J}$ denote the information-bearing signal and the jamming signal, $n_{a}$ is the additive white Gaussian noise (AWGN) at node *a* with $a\in \left\{D_{i,j}\right\}$, $i,j\in \left\{1,2\right\}$. Without loss of generality, the noise power spectral density is assumed the same everywhere within the network and is denoted as $N_{0}$, and $w_{ZF}$ is the normalized vector of the jamming operation.

Now, we will focus on the derivation of $w_{ZF}$. As pointed out previously, the jamming operation is introduced to increase the security of the transmission. Therefore, three purposes are expected to be achieved by this jamming operation. Firstly, the jamming operation will not affect the receiving performance of the legitimate information receiver. Secondly, the interference received at the eavesdropper should be maximized, so that the eavesdropper is confused as much as possible. Thirdly, the interference received at the legitimate energy receiver should be maximized, so that the legitimate energy receiver could collect as much energy as possible from the jamming signal. Mathematically, the above three ideas can be achieved by Equations (4)–(6), respectively,

(4) $$\left|h_{D_{3-i^{*}}D_{i^{*},j^{*}}}^{H}w_{ZF}\right|=0,$$

(5) $$\max_{w_{ZF}}\left|h_{D_{3-i^{*}}E}^{H}w_{ZF}\right|,$$

(6) $$\max_{w_{ZF}}\left|h_{D_{3-i^{*}}D_{i^{*},3-j^{*}}}^{H}w_{ZF}\right|,$$

where $h_{D_{3-i^{*}}E}\in C^{2\times 1}$ represents the channel coefficient vector from $D_{3-i^{*}}$ to *E*. In addition, recall that the jamming vector is normalized, hence we have

(7) $$∥w_{ZF}∥=1.$$

Unfortunately, due to the passive eavesdropping assumption, the eavesdropper’s CSI, $h_{D_{3-i^{*}}E}^{H}$, is unavailable at the legitimate network, thus it is not possible to maximize the confusing effect to the eavesdropper. As a result, we will try to find an appropriate $w_{ZF}$ so that Labels (4), (6), and (7) are all satisfied, namely

(8) $$\begin{array}{c} \max_{w_{ZF}}\left|h_{D_{3-i^{*}}D_{i^{*},3-j^{*}}}^{H}w_{ZF}\right|\\ s.t.\left|h_{D_{3-i^{*}}D_{i^{*},j^{*}}}^{H}w_{ZF}\right|=0,∥w_{ZF}∥=1. \end{array}$$

According to [25,26], the solution of Label (8) can be given by

(9) $$w_{ZF}=\frac{Th_{D_{3-i^{*}}D_{i^{*},3-j^{*}}}}{∥Th_{D_{3-i^{*}}D_{i^{*},3-j^{*}}}∥},$$

where T is the projection idempotent matrix with rank 1, which is given by

(10) $$T=I-h_{D_{3-i^{*}}D_{i^{*},j^{*}}}{\left(h_{D_{3-i^{*}}D_{i^{*},j^{*}}}^{H}h_{D_{3-i^{*}}D_{i^{*},j^{*}}}\right)}^{-1}h_{D_{3-i^{*}}D_{i^{*},j^{*}}}^{H}.$$

It is easy to see from Labels (9) and (10) that $w_{ZF}$ is chosen from the null space of the channel direction of $h_{D_{3-i^{*}}D_{i^{*},j^{*}}}$ so that the information receiving process is not interfered [27,28].

**Figure 2:**
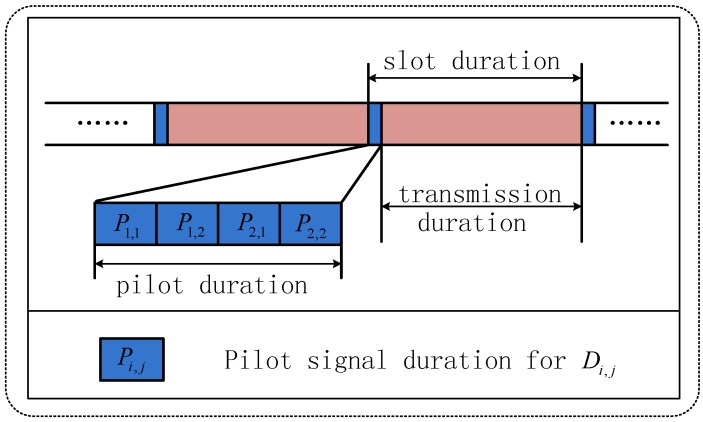
Time slot structure for user/antenna selection.

As can be observed, the CSI knowledge is required for both the selection process and ZF jamming operation. Now, we will elaborate on how this knowledge is obtained. As shown in Figure 2, each time slot could be divided into two parts, namely the pilot duration and the transmission duration. In order to select the best antenna for IR, the antenna $D_{i,j}$ (i,j∈{1,2}) will send pilot signals during its pilot duration $P_{i,j}$, which can be exploited for channel estimation by *S* and the other sensor, which acts as a jammer. Hence, *S* is able to obtain the knowledge of $h_{SD_{i,j}}$ for user/antenna selection, and the sensor that acts as jammer can derive the knowledge of $h_{D_{3-i^{*}}D_{i^{*},3-j^{*}}}$ and $h_{D_{3-i^{*}}D_{i^{*},j^{*}}}$ to construct the ZF jamming vector.

According to Labels (2) and (8), the receiving signal-to-noise ratio (SNR) for information receiving is given by

(11) $$\gamma_{D}=\frac{P_{S}}{N_{0}}\max_{i,j\in \left\{1,2\right\}}\;\left(g_{SD_{i,j}}\right).$$

At the same time, the amount of collected energy is expressed as

(12) $$\varepsilon_{D}=\eta T_{0}{\left|y_{D_{i^{*},3-j^{*}}}\right|}^{2},$$

and denoting $U=h_{D_{3-i^{*}}D_{i^{*},3-j^{*}}}^{H}w_{ZF}$. According to (Lemma 2 [25]), (Lemma 2 [26]), (Equation (30) [27]), (Equation (52) [28]), the CDF of $U=h_{D_{3-i^{*}}D_{i^{*},3-j^{*}}}^{H}w_{ZF}$ is a chi-square random variable with $2(L_{s}-1)$ degrees of freedom, namely *U* ~ $\chi^{2}\left(2\left(L_{s}-1\right)\right)$, where $L_{s}$ represents the number of ZF antennae. In this paper, we have $L_{s}=2$. Hence, *U* ~ $\chi^{2}\left(2\right)$, which degenerates into exponentially distributed random variables, namely we have

(13) $$f_{U}\left(u\right)=\frac{1}{{\bar{\gamma }}_{DD}}e^{-\frac{u}{{\bar{\gamma }}_{DD}}},$$

where ${\bar{\gamma }}_{ab}=E\left[g_{ab}\right]$ represents the average channel gain of link a−b, $a,b\epsilon \left\{S,D,E\right\}$. Specifically, ${\bar{\gamma }}_{DD}$ represents the average channel gain between the two sensors. By using the above result, Equation (12) can be readily calculated as (We note that little energy can be harvested from the AWGN in practice, hence is neglected in this paper [14,29].)

(14) $$\varepsilon_{D}=\eta T_{0}\left(P_{S}{\left|h_{SD_{i^{*},3-j^{*}}}\right|}^{2}+P_{J}{\left|h_{D_{3-i^{*}}D_{i^{*},3-j^{*}}}^{H}w_{ZF}\right|}^{2}\right),$$

where η is the energy conversion efficiency. Hence, in order to keep the energy balance at the sensors in the long run, the jamming power can be chosen as (We note that an energy outage would occur by adopting the approach in this paper. Although the probability to occur this can be proven to be very low by the simulations when an appropriate initial energy can be provided, it still should be considered for accurate analysis. In fact, by modeling battery as an energy queue would be a good method to give out the accurate analysis, which however is beyond the scope of this paper and is left for the research in the future.)

(15) $$P_{J}=\frac{E\left[\varepsilon_{D}\right]}{T_{0}}=\eta \left(P_{S}{\bar{\gamma }}_{SD}+P_{J}{\bar{\gamma }}_{DD}\right).$$

It is readily to know that Label (15) yields to

(16) $$P_{J}=\frac{\eta {\bar{\gamma }}_{SD}P_{S}}{1-\eta {\bar{\gamma }}_{DD}}.$$

Similarly, the receiving signal at *E* is expressed as

(17) $$y_{E}=\sqrt{P_{S}}h_{SE}x_{S}+\;\;\sqrt{P_{J}}h_{D_{3-i^{*}}E}^{H}w_{ZF}x_{J}+n_{E}.$$

Thus, the receiving signal-to-interference-plus-noise ratio (SINR) at *E* is given by

(18) $$\gamma_{E}=\frac{P_{S}g_{SE}}{P_{J}{\left|h_{D_{3-i^{*}}E}^{H}w_{ZF}\right|}^{2}+N_{0}}.$$

## 3. Secrecy Performance Analysis

In this section, we focus our attention on the secrecy performance of the proposed scheme. Before delving into the details, we present the preliminary of the following two lemmas.

**Lemma** **1.** *The CDF and the PDF of $\gamma_{D}$ are given by*

(19) $$F_{\gamma_{D}}\left(x\right)={\left(1-e^{-\frac{N_{0}x}{P_{S}{\bar{\gamma }}_{SD}}}\right)}^{4},$$

(20) $$f_{\gamma_{D}}\left(x\right)=\frac{4N_{0}}{P_{S}{\bar{\gamma }}_{SD}}\sum_{k=0}^{3}\left(\begin{array}{c} 3\\ k \end{array}\right){\left(-1\right)}^{k}e^{-\frac{\left(k+1\right)N_{0}x}{P_{S}{\bar{\gamma }}_{SD}}}.$$

**Proof.**  According to Label (11) and referring to [5], Equation (19) is readily obtained. By taking the derivation operation, the PDF of $\gamma_{D}$ is derived as

(21) $$f_{\gamma_{D}}\left(x\right)=\frac{4N_{0}}{P_{S}{\bar{\gamma }}_{SD}}{\left(1-e^{-\frac{N_{0}x}{P_{S}{\bar{\gamma }}_{SD}}}\right)}^{3}e^{-\frac{N_{0}x}{P_{S}{\bar{\gamma }}_{SD}}}.$$

By using the binomial theorem (Equation (1.111) [30]) in Label (21), the result in Label (20) is readily obtained. ☐

**Lemma** **2.** *The CDF and PDF of $\gamma_{E}$ are given by*

(22) $$F_{\gamma_{E}}\left(x\right)=1-\frac{P_{S}{\bar{\gamma }}_{SE}}{P_{J}{\bar{\gamma }}_{DE}x+P_{S}{\bar{\gamma }}_{SE}}e^{-\frac{N_{0}x}{P_{S}{\bar{\gamma }}_{SE}}},$$

(23) $$f_{\gamma_{E}}\left(x\right)=\frac{P_{S}{\bar{\gamma }}_{SE}P_{J}{\bar{\gamma }}_{DE}}{{\left(P_{J}{\bar{\gamma }}_{DE}x+P_{S}{\bar{\gamma }}_{SE}\right)}^{2}}e^{-\frac{N_{0}x}{P_{S}{\bar{\gamma }}_{SE}}}+\frac{N_{0}\left(P_{J}{\bar{\gamma }}_{DE}x+P_{S}{\bar{\gamma }}_{SE}\right)}{{\left(P_{J}{\bar{\gamma }}_{DE}x+P_{S}{\bar{\gamma }}_{SE}\right)}^{2}}e^{-\frac{N_{0}x}{P_{S}{\bar{\gamma }}_{SE}}}.$$

**Proof.**  Mathematically, the CDF of $\gamma_{E}$ can be manipulated as $F_{\gamma_{E}}\left(x\right)=\mathrm{Pr}\left(\gamma_{E}<x\right)$, which can be rewritten as Label (24) according to Label (18)

(24) $$F_{\gamma_{E}}\left(x\right)=\int_{0}^{\infty }F_{g_{SE}}\left(\frac{\left(P_{J}v+N_{0}\right)x}{P_{S}}\right)f_{V}\left(v\right)dv,$$

where $V={\left|h_{D_{3-i^{*}}E}^{H}w_{ZF}\right|}^{2}$. As shown in Label (8), $w_{ZF}={\left[w_{1},w_{2}\right]}^{T}$ is a normalized vector, i.e., ${\left|w_{1}\right|}^{2}+{\left|w_{2}\right|}^{2}=1$. Thus, $\left|h_{D_{3-i^{*}}E}^{H}w_{ZF}\right|$ is a unitary transformation of $h_{D_{3-i^{*}}E}^{H}$. In addition, it is obvious that $w_{ZF}$ is independent of $h_{D_{3-i^{*}}E}^{H}$, and the two elements of $h_{D_{3-i^{*}}E}^{H}$, namely $h_{D_{3-i^{*},1}E}$ and $h_{D_{3-i^{*},2}E}$ are both Gaussian variables. In other words, we have $h_{D_{3-i^{*},1}E},h_{D_{3-i^{*},2}E}∼N\left(0,{\bar{\gamma }}_{DE}\right)$. Therefore, $w_{1}h_{D_{3-i^{*},1}E}∼N\left(0,{\left|w_{1}\right|}^{2}{\bar{\gamma }}_{DE}\right)$ and $w_{2}h_{D_{3-i^{*},2}E}∼N\left(0,{\left|w_{2}\right|}^{2}{\bar{\gamma }}_{DE}\right)$ can be concluded. Hence, $\left(w_{1}h_{D_{3-i^{*},1}E}+w_{2}h_{D_{3-i^{*},2}E}\right)∼N\left(0,\left({\left|w_{1}\right|}^{2}+{\left|w_{2}\right|}^{2}\right){\bar{\gamma }}_{DE}\right)=N\left(0,{\bar{\gamma }}_{DE}\right)$, which means that $V={\left|w_{1}h_{D_{3-i^{*},1}E}+w_{2}h_{D_{3-i^{*},2}E}\right|}^{2}$ is also an exponentially distributed random variables with the mean of ${\bar{\gamma }}_{DE}$. We note that *V* has the same distribution with $h_{D_{3-i^{*},1}E}$ and $h_{D_{3-i^{*},2}E}$, and this has verified the conclusion that the unitary transformation does not change the distribution of the transformed variables. As a result, we have

(25) $$f_{V}\left(v\right)=\frac{1}{{\bar{\gamma }}_{DE}}e^{-\frac{v}{{\bar{\gamma }}_{DE}}}.$$

In addition, the CDF of $g_{SE}$ is given by

(26) $$F_{g_{SE}}\left(x\right)=1-e^{-\frac{x}{{\bar{\gamma }}_{SE}}}.$$

Substituting Labels (25) and (26) into Label (24) and after some calculations, the CDF of $\gamma_{E}$ is easily obtained as in Label (22). By taking the derivation operation in Label (22), we finally derive the result in Label (23). ☐

### 3.1. Secrecy Outage Probability

The secrecy outage probability is defined as the probability that the instantaneous secrecy capacity falls below a predefined secrecy rate $R_{s}$ (The design of $R_{s}$ falls into the construction of the wiretap coding, which has been elaborated abundantly in the literature [31,32], and thus is omitted in this paper.), which can be equivalently expressed as [5,28]

(27) $$P_{out}=\int_{0}^{\infty }\;\;\;F_{\gamma_{D}}\left(\gamma_{th}^{s}\;+\;\gamma_{th}^{s}y-\;1\right)f_{\gamma_{E}}\left(y\right)dy,$$

where $\gamma_{th}^{s}=2^{R_{s}}$.

**Theorem** **1.** *The secrecy outage probability for the proposed system is given by*

(28) $$P_{out}\left(\gamma_{th}^{s}\right)=\sum_{k=0}^{4}\left(\begin{array}{c} 4\\ k \end{array}\right){\left(-1\right)}^{k}e^{-\frac{kN_{0}\left(\gamma_{th}^{s}-1\right)}{P_{S}{\bar{\gamma }}_{SD}}}\left(I_{1,k}+I_{2,k}\right),$$

*where*

(29) $$I_{1,k}=1+\frac{N_{0}}{P_{J}{\bar{\gamma }}_{DE}{\bar{\gamma }}_{SD}}\left({\bar{\gamma }}_{SD}+k\gamma_{th}^{s}{\bar{\gamma }}_{SE}\right)\exp(\frac{N_{0}{\bar{\gamma }}_{SD}+kN_{0}\gamma_{th}^{s}{\bar{\gamma }}_{SE}}{P_{J}{\bar{\gamma }}_{DE}{\bar{\gamma }}_{SD}})Ei\left(-\;\frac{N_{0}{\bar{\gamma }}_{SD}\;\;+\;\;kN_{0}\gamma_{th}^{s}{\bar{\gamma }}_{SE}}{P_{J}{\bar{\gamma }}_{DE}{\bar{\gamma }}_{SD}}\;\;\right),$$

(30) $$I_{2,k}=-\frac{N_{0}}{P_{J}{\bar{\gamma }}_{DE}}\exp\left(\frac{N_{0}{\bar{\gamma }}_{SD}+kN_{0}\gamma_{th}^{s}{\bar{\gamma }}_{SE}}{P_{J}{\bar{\gamma }}_{DE}{\bar{\gamma }}_{SD}}\right)Ei\left(-\frac{N_{0}{\bar{\gamma }}_{SD}+kN_{0}\gamma_{th}^{s}{\bar{\gamma }}_{SE}}{P_{J}{\bar{\gamma }}_{DE}{\bar{\gamma }}_{SD}}\right).$$

**Proof.**  Replacing *x* with $\left(\gamma_{th}^{s}+\gamma_{th}^{s}y-1\right)$ in Label (19), we derive

(31) $$F_{\gamma_{D}}\left(\gamma_{th}^{s}+\gamma_{th}^{s}y-1\right)={\left(1-\exp\left(-\frac{N_{0}\left(\gamma_{th}^{s}+\gamma_{th}^{s}y-1\right)}{P_{S}{\bar{\gamma }}_{SD}}\right)\right)}^{4}.$$

With the help of binomial theorem, Label (31) can be rewritten as

(32) $$F_{\gamma_{D}}\left(\gamma_{th}^{s}+\gamma_{th}^{s}y-1\right)=\sum_{k=0}^{4}\left(\begin{array}{c} 4\\ k \end{array}\right){\left(-1\right)}^{k}\exp\left(-\frac{kN_{0}\left(\gamma_{th}^{s}+\gamma_{th}^{s}y-1\right)}{P_{S}{\bar{\gamma }}_{SD}}\right).$$

By substituting Labels (23) and (32) into Label (27), and letting $t=y-\frac{1-\gamma_{th}^{s}}{\gamma_{th}^{s}}$, we obtain

(33) $$P_{out}=\sum_{k=0}^{4}\left(\begin{array}{c} 4\\ k \end{array}\right){\left(-1\right)}^{k}\exp\left(-\frac{kN_{0}\left(\gamma_{th}^{s}-1\right)}{P_{S}{\bar{\gamma }}_{SD}}\right)\left(I_{1,k}+I_{2,k}\right),$$

where

(34) $$I_{1,k}=\int_{0}^{\infty }\frac{P_{S}{\bar{\gamma }}_{SE}P_{J}{\bar{\gamma }}_{DE}}{{\left(P_{J}{\bar{\gamma }}_{DE}y+P_{S}{\bar{\gamma }}_{SE}\right)}^{2}}\exp\left(-\frac{N_{0}{\bar{\gamma }}_{SD}+kN_{0}\gamma_{th}^{s}{\bar{\gamma }}_{SE}}{P_{S}{\bar{\gamma }}_{SE}{\bar{\gamma }}_{SD}}y\right)dy,$$

(35) $$I_{2,k}=\int_{0}^{\infty }\frac{N_{0}}{P_{J}{\bar{\gamma }}_{DE}y+P_{S}{\bar{\gamma }}_{SE}}\exp\left(-\frac{N_{0}{\bar{\gamma }}_{SD}+kN_{0}\gamma_{th}^{s}{\bar{\gamma }}_{SE}}{P_{S}{\bar{\gamma }}_{SE}{\bar{\gamma }}_{SD}}y\right)dy.$$

By using ∫0∞e−pxe−pxa+x2a+x2dx=peapEi−ap+11aa (Equation (3.353.3) [30]), Label (34) leads to Label (29). Similarly, by using ∫0∞e−μxe−μxx+βx+βdx=−eβμEi−βμ (Equation (3.352.4) [30]), Label (35) results in Label (30). ☐

### 3.2. Secrecy Throughput

The secrecy throughput can be defined as the secrecy rate multiplied by the probability of a reliable and secure transmission, which is mathematically written as [33]

(36) $$\varsigma =R_{s}\left(1-P_{out}\right).$$

By substituting the result in Theorem 1 into Label (36), the secrecy throughput can be easily deduced.

## 4. Simulation Results and Discussion

In this section, some representative simulations are provided to examine the impacts of the system parameters on the cooperative zero-forcing jamming scheme for the two-user SWIPT networks. The transmit SNR is defined as $\gamma_{S}=P_{S}/N_{0}$. As can be readily observed, the theoretical results are in exact agreement with the simulations, validating the correctness of the analysis. Without loss of generality, we set $N_{0}=1$.

**Figure 3:**
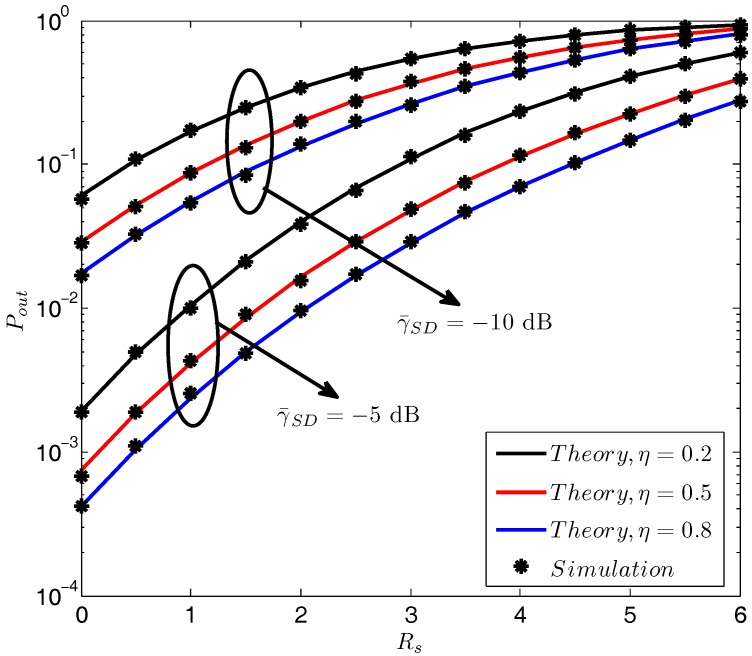
$P_{out}$ vs. $R_{s}$ with different ${\bar{\gamma }}_{SD}$ and η. $\gamma_{S}=30$ dB, ${\bar{\gamma }}_{SE}=-10$ dB, ${\bar{\gamma }}_{DE}=-10$ dB, ${\bar{\gamma }}_{DD}=-5$ dB.

Figure 3 illustrates the secrecy outage probability $P_{out}$ versus the secrecy rate $R_{s}$ with different ${\bar{\gamma }}_{SD}$ and η. As it is shown, the secrecy outage probability monotonically increases with the growth of $R_{s}$. In addition, we see from Figure 3 that larger ${\bar{\gamma }}_{SD}$ and η both lead to a better secrecy outage performance. It is not difficult for comprehension because a larger secrecy rate is much harder to support for a given channel condition, thus leading to a greater secrecy outage. In addition, a larger ${\bar{\gamma }}_{SD}$ indicates a greater amount of harvested energy and a better receiving SNR performance at the receiver. In other words, by increasing ${\bar{\gamma }}_{SD}$, the receiving SNR performance at the receiver is promoted while the receiving SNR performance at the eavesdropper becomes poor because a larger jamming power could be provided. Furthermore, although it is not a benefit to the receiver by increasing η, it still improves the amount of harvested energy, and thus can confuse the eavesdropper better. Therefore, increasing ${\bar{\gamma }}_{SD}$ and η both contribute to a better secrecy outage performance.

**Figure 4:**
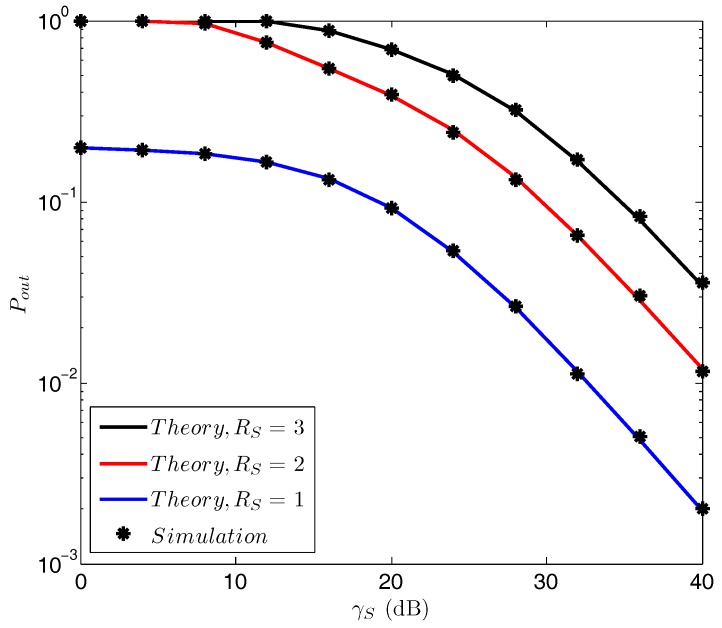
$P_{out}$ vs. $\gamma_{S}$ with various $R_{s}$. ${\bar{\gamma }}_{SD}=-10$ dB, ${\bar{\gamma }}_{SE}=-10$ dB, ${\bar{\gamma }}_{DE}=-10$ dB, ${\bar{\gamma }}_{DD}=-5$ dB, η=0.8.

Figure 4 plots the secrecy outage probability $P_{out}$ versus the transmit SNR $\gamma_{S}$ for various $R_{s}$. As can be seen, the secrecy outage probability is a monotonically increasing function with $R_{s}$, which coincides with the finding in Figure 3. In addition, a lower secrecy outage probability is observed when a larger $\gamma_{S}$ is provided, regardless of the value of $R_{s}$. As a matter of fact, the changing of $\gamma_{S}$ will have two effects on the performance. On the one hand, increasing $\gamma_{S}$ will benefit the receiving performance of both the receiver’s and the eavesdropper’s. One the other hand, the performance of the eavesdropper will be degraded by the jamming operation while the performance of the information receiver will not. We note that, when a larger $\gamma_{S}$ is provided, the confusing effect to the eavesdropper will become better because more energy will be harvested, so that the user can jam the eavesdropper at a greater power. Overall, the receiving performance of the information receiver is much more improved than that of the eavesdropper. Therefore, the secrecy outage probability becomes a decreasing function with $\gamma_{S}$.

**Figure 5:**
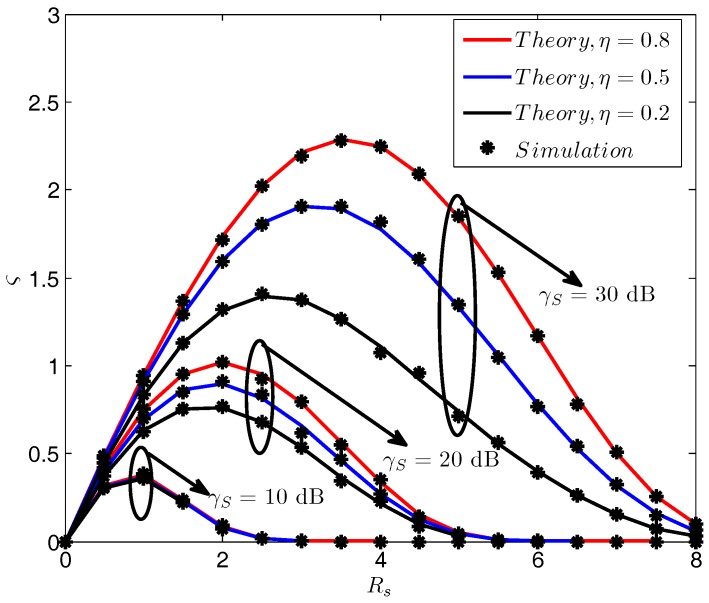
$P_{out}$ vs. $R_{s}$ with different $\gamma_{S}$ and η. $\gamma_{S}=30$ dB, ${\bar{\gamma }}_{SD}=-10$ dB, ${\bar{\gamma }}_{SE}=-10$ dB, ${\bar{\gamma }}_{DE}=-10$ dB, ${\bar{\gamma }}_{DD}=-5$ dB.

Figure 5 compares the secrecy throughput ς versus the secrecy rate $R_{s}$ with different $\gamma_{S}$ and η. It is shown that the secrecy throughput improves significantly when a larger $\gamma_{S}$ is provided. Moreover, it is also beneficial to boost the secrecy throughput if η could be increased. In addition, this is especially useful in the high SNR region, as the enhancement of secrecy throughput is much more notable when $\gamma_{S}$ is large. Furthermore, it is noted that the variation tendency of the secrecy throughput in each line indicates the existence of an optimum $R_{s}$ which can maximize the secrecy throughput. This phenomenon is comprehensible. On the one hand, increasing $R_{s}$ will directly contribute to the enhancement of secrecy throughput, as more secrecy message is transmitted. On the other hand, a larger secrecy rate will also lead to a greater secrecy outage probability, which will result in the decline of the secrecy throughput. As a result, an optimum $R_{s}$ that maximizes the secrecy throughput is observed.

## 5. Conclusions

This paper presented a novel secure transmission scheme for the two-user SWIPT sensor networks where the cooperative zero-forcing jamming was conducted to confuse the eavesdropper. It is highlighted that the cooperative jamming does not require any energy of the sensors due to the SWIPT operation, and thus can be well applied to the energy-constrained wireless sensor networks. The exact closed-form expressions of the secrecy outage probability and the secrecy throughout were derived, which depicted the impacts of the system parameters on the system secrecy performance intuitively. The results illustrated that the secrecy outage probability monotonically increases with the growth of $R_{s}$, and monotonically decreases with the increase of $\gamma_{S}$ and η. Moreover, the secrecy throughput could be further boosted if η increases, which is especially notable when $\gamma_{S}$ is large enough. In addition, it was indicated that an optimum value of $R_{s}$ maximizing the secrecy throughput exists, which also grows with the increase of $\gamma_{S}$. All of the findings are of great importance in guiding the secure design of practical wireless sensor networks.

## Figures and Tables

**Table 1:** Working mode of the sensors and antennas $\left(i^{*},j^{*}\in \left\{1,2\right\}\right)$.

Sensor	Antenna	Working Mode
$D_{i^{*}}$	$D_{i^{*},j^{*}}$	Information receiving
	$D_{i^{*},3-j^{*}}$	Energy harvesting
$D_{3-i^{*}}$	$D_{3-i^{*},j^{*}}$	Zero-forcing jamming
	$D_{3-i^{*},3-j^{*}}$	
